# Emergence of new recombinant noroviruses GII.p16-GII.4 and GII.p16-GII.2, France, winter 2016 to 2017

**DOI:** 10.2807/1560-7917.ES.2017.22.15.30508

**Published:** 2017-04-13

**Authors:** Maxime Bidalot, Lucie Théry, Jérôme Kaplon, Alexis De Rougemont, Katia Ambert-Balay

**Affiliations:** 1 National Reference Centre for Gastroenteritis Viruses, Laboratory of Biology and Pathology, University Hospital Dijon Bourgogne, Dijon, France; 2 University Bourgogne Franche-Comté, AgroSup Dijon, PAM UMR A 02.102, Dijon, France

**Keywords:** Norovirus, recombinants, co-circulation, emergence, gastroenteritis

## Abstract

An early increase in outbreaks of norovirus gastroenteritis characterised at the French National Reference Centre occurred this winter season. They were concurrent with an unusual pattern of circulating strains, with three predominant genotypes: the re-emergent variant GII.P4 2009-GII.4 2012 found in 28% of norovirus outbreaks and two new emergent recombinant strains GII.P16-GII.4 2012 and GII.P16-GII.2 never before observed in France, found in 24% and 14% of norovirus outbreaks, respectively.

We report an early increase in norovirus (NoV) gastroenteritis outbreaks investigated during this 2016/17 season at the French National Reference Centre for Gastroenteritis Viruses (NRCgev), compared with the previous season (Figure 1). Molecular characterisation and phylogenetic analysis of the strains responsible for these outbreaks showed that three predominant genotypes were co-circulating, including two new emergent recombinant strains never before observed in France.

**Figure 1:**
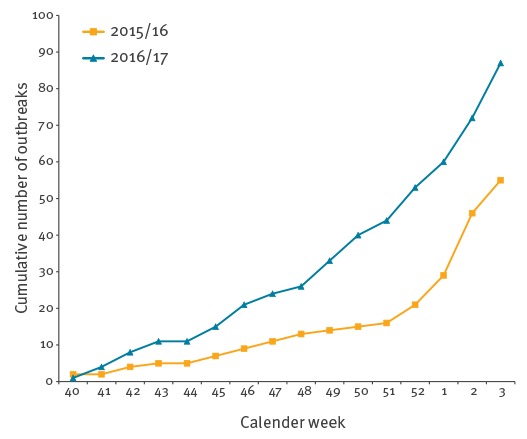
Cumulative number of norovirus outbreaks investigated at the French National Reference Centre for Gastroenteritis Viruses, France, week 40 to week 3, 2015/16 (n = 55) and 2016/17 (n = 87)

## Laboratory investigation

From week 40 in 2016 to week 3 in 2017, 350 stool samples corresponding to 114 gastroenteritis outbreaks were investigated at the French NRCgev. NoV detection was performed by real-time RT-PCR as previously described [1]. A total of 222 stool samples, corresponding to 87 outbreaks (76%), were positive for norovirus. In comparison, during the same period in 2015/16, 55 of 76 outbreaks (72%) had been positive for norovirus (Figure 1). Interestingly, the increase in norovirus-positive outbreaks started earlier this winter season than in the previous season.

**Figure 2:**
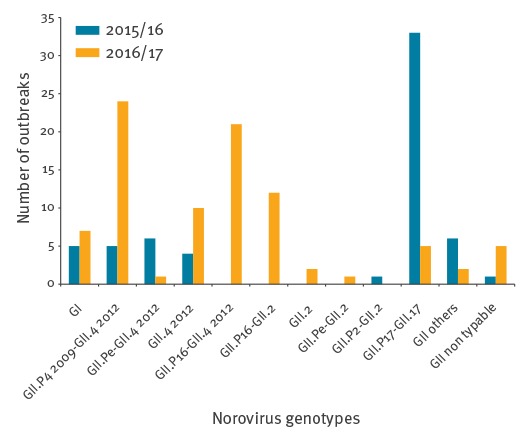
Diversity of norovirus genotypes found at the French National Reference Centre for Gastroenteritis Viruses, France, week 40 to week 3, 2015/16 (n = 61) and 2016/17 (n = 90)

Two to three norovirus-positive specimens from each positive outbreak were genotyped as previously described [1], by sequencing a fragment of the RNA polymerase gene (open reading frame (ORF) 1) and a fragment of the capsid gene (ORF2). Genotype was determined using the Norovirus Genotyping Tool version 1.0 [2]. Furthermore, for a selection of samples for which ORF1 and ORF2 presented different genotypes, direct sequencing of a 1,112 bp region spanning the 3’ end of ORF1 and the 5’ end of ORF2 was performed to confirm the recombination status. Amplification was performed using the primer set JV12/G2SKR. ORF1-ORF2 amplification and sequencing confirmed a recombination event for 27 samples. Nucleotide sequences of these samples were submitted to the GenBank database under accession numbers KY817495 to KY817521. Figure 2 presents the diversity of NoV genotypes found in the current and the previous season, between week 40 and week 3.

Three genotypes were predominant this season: the variant GII.P4 2009-GII.4 2012 found in 24 of 87 norovirus outbreaks (28%), the recombinant GII.P16-GII.4 2012 in 21 outbreaks (24%), and the recombinant GII.P16-GII.2 in 12 outbreaks (14%). Furthermore, 12 strains could only be partially characterised, 10 with a GII.4 2012 capsid and two with a GII.2 capsid. In comparison, one single genotype GII.P17-GII.17 had predominated during the 2015/16 season (54% of outbreaks), a genotype that was rarely found at the beginning of the current season (n = 5; 6%).

**Figure 3:**
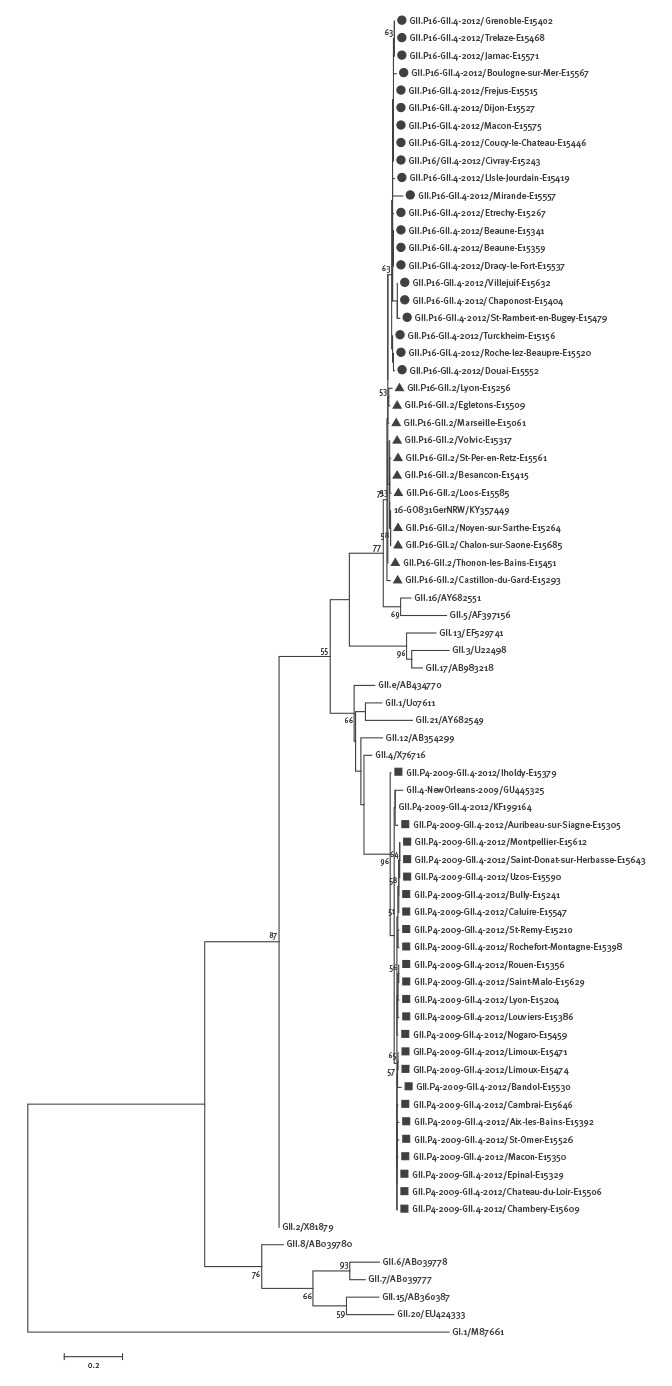
Phylogenetic tree based on the partial nucleotide sequences (287 bp) of the norovirus polymerase gene

**Figure 4:**
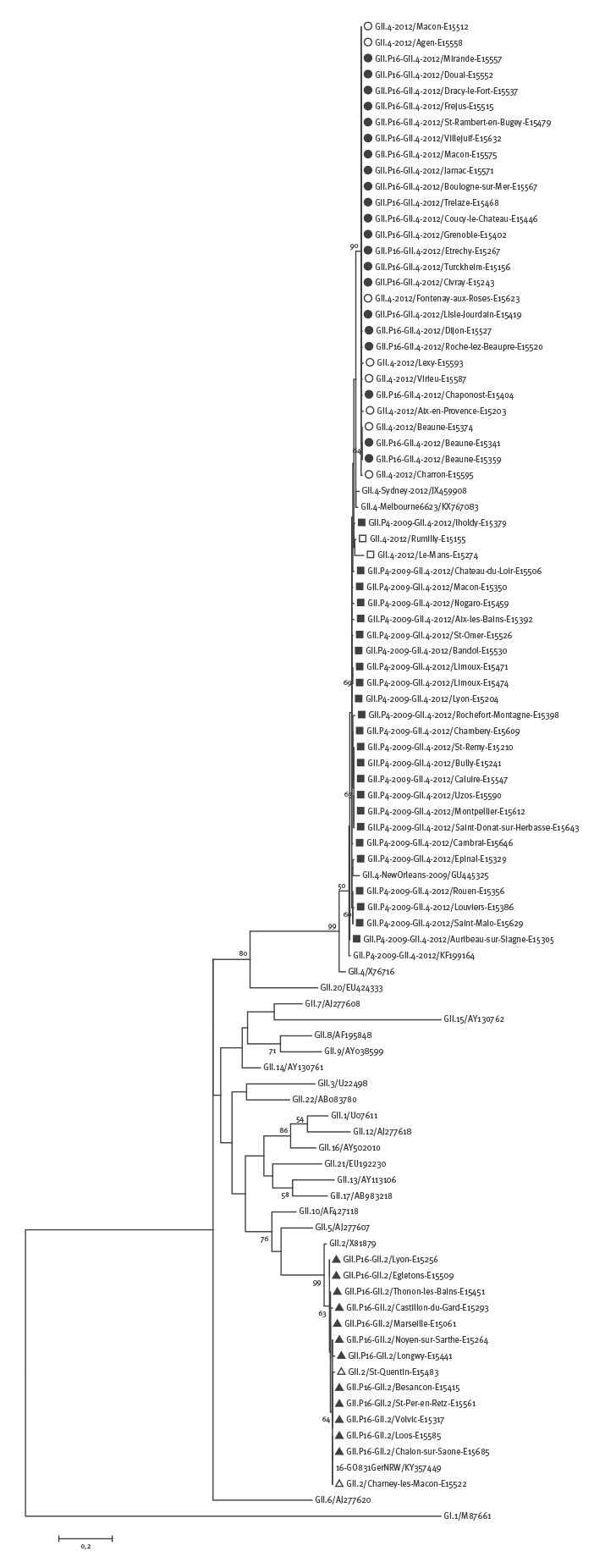
Phylogenetic tree based on the partial nucleotide sequences (266 bp) of the norovirus capsid gene

Phylogenetic analysis showed that all the GII.P4 2009-GII.4 2012 strains found in this study clustered with the strain GII.P4 2009-GII.4 2012 (GenBank KF199164) found in Denmark in March 2013 [3], in both the polymerase and capsid regions (Figures 3 and 4).

Phylogeny was reconstructed using the maximum-likelihood method implemented in MEGA6 [11] with the Kimura 2-parameter substitution model (i.e. best nucleotide substitution model for the dataset). The number of substitutions per site is indicated by the scale bar. Bootstrap values were calculated for 500 replicates and are indicated at each node when ≥ 50%. French norovirus strains from this study are labelled as follows: full circles for GII.P16-GII.4, full triangles for GII.P16-GII.2 and full squares for GII.P4-GII.4.

Phylogeny was reconstructed using the maximum-likelihood method implemented in MEGA6 [11] with the Kimura 2-parameter substitution model (i.e. best nucleotide substitution model for the dataset). The number of substitutions per site is indicated by the scale bar. Bootstrap values were calculated for 500 replicates and are indicated at each node when ≥ 50%. French norovirus strains from this study are labelled as follows: full and empty circles, respectively, for GII.P16-GII.4 and GII.4 strains probably bearing a GII.P16 genotype; full and empty triangles, respectively, for GII.P16-GII.2 and GII.2 strains probably bearing a GII.P16 genotype; full and empty squares, respectively, for GII.P4-GII.4 and GII.4 strains probably bearing a GII.P4 genotype.

They also clustered in the sequenced capsid fragment with the reference strain GII.4 Sydney 2012 (JX459908) and with the GII.4 Melbourne 6623 (KX767083) found in Australia in June 2016 [4]. The polymerase region of the GII.P16-GII.4 2012 strains and GII.P16-GII.2 strains were all closely related to the GII.P16-GII.16 strain VannesL23/1999/FR (AY682551), but interestingly, they separated in two distinct clusters (Figure 3). Of note, the polymerase sequence of the new recombinant GII.P16-GII.2 GO831GerNRW (KY357449) found in Germany this winter season [5] appeared in the same cluster as the French GII.P16-GII.2 strains.

In the capsid region, the GII.P16-GII.2 strains clustered with the reference strain Melksham/1994/UK (X81879) and were closely related to the recombinant GII.P16-GII.2 GO831GerNRW (KY357449) (Figure 4). Of note, two GII.2 capsid sequences for which no polymerase sequence could be determined also appeared in the same cluster. The capsid sequences of the GII.P16-GII.4 2012 strains were closely related to the reference strains GII.4 Sydney 2012, but interestingly, they segregated in a clearly distinct cluster from the GII.P4 2009-GII.4 2012 (Figure 4). It has to be noted that of the 10 GII.4 2012 strains for which polymerase gene amplification and sequencing failed, eight clustered with the GII.P16-GII.4 2012 strains and two clustered with the GII.P4 2009-GII.4 2012 strains, suggesting that the former may bear a GII.16 polymerase genotype while the latter may bear a GII.4 polymerase genotype.

## Discussion

We observed an unusual co-circulation of three norovirus strains this winter season, including two emergent recombinant strains never before detected in France. The co-circulation of two strains has occasionally been observed, such as the 2006a with the 2006b variant, but this was geographically and temporally limited [6]. Usually and for more than 20 years, gastroenteritis epidemics reported all over the world have been linked to a single predominant strain, principally a succession every two to three years of GII.4 genotypic variants, including US95/96 1996, Farmington Hills 2002, Hunter 2004, Den Haag 2006b, New Orleans 2009 and Sydney 2012 [6-8]. Unexpectedly, in the winter of 2014/15, a GII.17 strain emerged in Asia and then replaced the previously predominant GII.4 Sydney 2012 [9]. In France, the GII.17 strain became predominant in the winter of 2015/16 (data not shown). 

One of this season’s predominant strains, the variant GII.P4 2009-GII.4 2012, had already been detected in France during the seasons 2012/13, 2013/14 and 2014/15, at a time when the variant Sydney 2012 largely predominated, and to a lesser extent in 2015/16, when the strain GII.17 predominated. This variant was described in Denmark and Italy during the season 2012/13 [3,10] and more recently in Australia in August 2015 and as an altered version in June 2016 [4]. Interestingly, the Australian authors suggested that this current recombinant strain could have the potential to become a pandemic variant [4]. However, the partial sequences of the capsid gene obtained in our laboratory do not provide enough information to differentiate between the 2012/13 variant and the derivative, and further molecular investigations are needed.

The two recombinant strains GII.P16-GII.4 2012 and GII.P16-GII.2 had never been observed in France before this winter season and have to our knowledge never been reported as major strains responsible for outbreaks in any country before this season. Although they were circulating concurrently this season in Germany, the reported pattern of circulating strains was different from what was observed in France [5]. Indeed, the GII.P16-GII.2 was the predominant strain responsible for 42% of outbreaks in Germany, far ahead of the variant GII.P4 2009-GII.4 2012 (10%) and the recombinant GII.P16-GII.4 2012 (10%), while in France it was third after GII.P4 2009-GII.4 2012 and GII.P16-GII.4. The reasons for these differences are unclear. One could be the setting of the outbreaks, since the majority of the investigated outbreaks in France occurred in nursing homes (73%), while in Germany, they occurred mainly in childcare facilities (56% vs 17% in nursing homes). However, a complete assessment will be necessary at the end of the season to draw any conclusions about the pattern of predominant circulating strains in France, since the data reported here concern the outbreaks investigated between week 40 in 2016 and week 3 in 2017 and the gastroenteritis outbreak season is not yet over. Already, according to the phylogenetic analysis of the capsid sequences, it seems that GII.P16-GII.4 strains could be more prevalent than the GII.P4 2009-GII.4 2012 variants. Further molecular and epidemiological investigations are needed to confirm this tendency.
